# Potential of Polycyclic Aromatic Hydrocarbon-Degrading Bacterial Isolates to Contribute to Soil Fertility

**DOI:** 10.1155/2016/5798593

**Published:** 2016-09-28

**Authors:** Maryam Bello-Akinosho, Rosina Makofane, Rasheed Adeleke, Mapitsi Thantsha, Michael Pillay, George Johannes Chirima

**Affiliations:** ^1^Department of Microbiology and Plant Pathology, University of Pretoria, Lynnwood Road, Hatfield, Pretoria 0002, South Africa; ^2^Agricultural Research Council-Institute for Soil, Climate and Water (ARC-ISCW), 600 Belvedere Street, Arcadia, Pretoria 0001, South Africa; ^3^Department of Biotechnology, Vaal University of Technology, Vanderbijlpark 1900, South Africa; ^4^Unit for Environmental Science and Management, North-West University, Potchefstroom Campus, Potchefstroom 2520, South Africa; ^5^Centre for African Ecology, School of Animal, Plant and Environmental Sciences, University of the Witwatersrand, Wits 2050, South Africa

## Abstract

Restoration of polycyclic aromatic hydrocarbon- (PAH-) polluted sites is presently a major challenge in agroforestry. Consequently, microorganisms with PAH-degradation ability and soil fertility improvement attributes are sought after in order to achieve sustainable remediation of polluted sites. This study isolated PAH-degrading bacteria from enriched cultures of spent automobile engine-oil polluted soil. Isolates' partial 16S rRNA genes were sequenced and taxonomically classified. Isolates were further screened for their soil fertility attributes such as phosphate solubilization, atmospheric nitrogen fixation, and indoleacetic acid (IAA) production. A total of 44 isolates were obtained and belong to the genera *Acinetobacter*, *Arthrobacter*, *Bacillus*,* Flavobacterium*,* Microbacterium*,* Ochrobactrum*,* Pseudomonas*, *Pseudoxanthomonas*,* Rhodococcus*, and* Stenotrophomonas*. Data analysed by principal component analysis showed the* Bacillus* and *Ochrobactrum* isolates displayed outstanding IAA production. Generalized linear modelling statistical approaches were applied to evaluate the contribution of the four most represented genera (*Pseudomonas*,* Acinetobacter*,* Arthrobacter*, and *Rhodococcus*) to soil fertility. The* Pseudomonas* isolates were the most promising in all three soil fertility enhancement traits evaluated and all isolates showed potential for one or more of the attributes evaluated. These findings demonstrate a clear potential of the isolates to participate in restorative bioremediation of polluted soil, which will enhance sustainable agricultural production and environmental protection.

## 1. Introduction

Certain soil nutrients are vital for the growth and survival of plants in either small or large quantities. Those essential nutrients needed in large quantities are called macronutrients and include nitrogen, phosphorus, and potassium [1] but are, however, present in soil in limiting amounts [2] which therefore necessitate the addition of inorganic fertilizers such as NPK [1, 3]. A large portion of the inorganic phosphorus in NPK is, however, rapidly immobilized and becomes unavailable for uptake by plants [4]. To overcome this, some soil bacteria are known to possess abilities for converting immobilized forms of phosphorus to readily available forms. They also fix atmospheric nitrogen to ammonia and subsequently nitrates and produce and modulate indoleacetic acid (indole-3-acetic acid, IAA) in the form of auxin [5]. They are usually associated with plant root or soil and are referred to as Plant Growth Promoting Rhizobacteria (PGPR) [6–8]. Bacteria with these abilities are indispensable in improving soil fertility [9, 10]. Thus, PGPR are envisaged to be a suitable alternative to chemical fertilizers [7, 9, 11].

Agricultural land may be exposed to pollution by polycyclic aromatic hydrocarbons (PAHs) via mining or when there is spillage of petroleum products. Polycyclic aromatic hydrocarbons are a ubiquitous and recalcitrant group of organic environmental pollutants [12], known for their hydrophobic nature, which greatly reduces their availability for degradation and promotes their persistence in soil. In spite of this, a number of bacterial genera have been reported to possess specific mechanisms for degradation of PAHs [13–16]. This process is termed microbial bioremediation [17], which is an economic and ecofriendly way of soil remediation where bacteria degrade the hydrocarbons in soil to nontoxic or less toxic end products [17, 18]. The degradation ability is an adaptation mechanism and once adapted, the degrading microorganisms have accelerated metabolism, which necessitates nutrient stimulation, which can be overcome by microorganisms with innate characteristics of nutrient addition to the soil. This ensures sustainability of the long-term process of bioremediation.

The ultimate aim of any bioremediation process is to restore polluted land to a prepollution state. Given the fact that contaminated soils are often nutrient poor [19], bacteria, which are able to simultaneously degrade PAHs and enhance soil fertility, are valuable as bioaugmentation strains in phytoremediation [18]. Such combination of attributes is indispensable for sustainable agricultural production and environmental protection particularly in the mining and agroforestry sectors. Very few studies have been reported in this respect [18, 20]. The present study aimed to evaluate,* in vitro*, the potential roles of PAH-degrading isolates to solubilize phosphates, fix atmospheric nitrogen, and produce IAA as an exploration of their ability to contribute to soil fertility and restorative bioremediation.

## 2. Materials and Methods

### 2.1. Soil Preparation

Agricultural soil with no history of pollution was collected from a research farm of the Agricultural Research Council, Vegetable and Ornamental Plant Institute, Pretoria, South Africa (25°36′04.2′′S; 28°22′01.3′′E). The soil is of alluvial origin and classified as an Oakleaf soil form [21]. Sampling was done from the topsoil (0–250 mm). The soil was ground and sieved through a 2 mm sieve to remove solid particles and minimize heterogeneity. Parameters including particle size distribution, pH, cation exchange capacity (CEC), and nutrient levels were analysed before contamination with spent engine oil. These are shown in Table 1.

Contamination with spent automobile engine oil, whose PAH content was previously determined, was performed by mixing 0 g (control), 5 g, and 50 g of oil per kilogram of soil. The contaminated soil was biostimulated with vermicompost obtained from the vermicompost laboratory of the Agricultural Research Council, in Pretoria. Biostimulation of soil with vermicompost was done at 0% (control), 20%, and 40%, thus maintaining and boosting the indigenous soil microbes. Contamination was done prior to biostimulation to reflect the actual occurrence of events on contaminated sites. The experimental design was a factorial experiment.

### 2.2. Isolation of PAH-Degrading Bacteria

The methods of Hilyard et al. [22] were slightly modified for the isolation of PAH-degrading bacteria from soil samples, which were collected after 10 weeks of treatment. Pure cultures from various treatments of the artificial pollution were isolated after selective enrichment in Bushnell Haas (BH) broth [23] supplemented with three PAHs, naphthalene, phenanthrene, and fluoranthene, that served as the sole source of carbon. Briefly, 1 g of each soil treatment was aseptically transferred into 250 mL Erlenmeyer flasks containing 100 mL BH minimal medium supplemented with 25 mg of phenanthrene or fluoranthene or 50 mg of naphthalene. Two successive enrichments were done with 1% (v/v) culture fluid of the preceding enrichment as inoculum for the succeeding subculture at 3-week intervals. The cultures were incubated at 28°C with shaking at 175 rpm. Aliquots from the second and third enrichments were plated on BH agar plates and sprayed with the same PAH used in the enrichment. Colonies arising therefrom were subcultured in BH agar plates and sprayed with the same PAH. The PAH-degrading strains were selected based on the enhanced growth on plates with added PAH compared to the control plates without added PAH and they were stored in 50% glycerol at −80°C for future use.

### 2.3. Molecular Identification, Phylogenetic, and Community Analyses of Isolates

For bacterial identification, the 16S rRNA gene of pure isolates was amplified using primer sets 968R (5′-AAC GCG AAG AAC CTT AC-3′) and 1401F (5′-CGG TGT GTA CAA GAC CC-3′) [24] using a colony PCR approach. All PCR were performed in a thermal cycler T100*™* (Bio-Rad, USA) and each 20 *μ*L PCR reaction contained 0.2 *μ*M of each forward and reverse primers, 10 *μ*L of Thermo Scientific 2x Phusion flash High-Fidelity PCR Master Mix, colony template, and sterile nuclease-free water. The PCR conditions were 20 s of initial denaturation at 98°C, 30 cycles of 98°C for 20 s, 56°C for 30 s, 72°C for 30 s, and a final extension at 72°C for 5 mins.

Electrophoresis of 5 *μ*L amplicons and loading dye was done on an ethidium bromide stained 1% agarose gel and subsequently visualized with UV radiation in a Bio-Rad Gel Doc EZ Imager (Gel Doc, Bio-Rad, USA). Amplicon clean-up was done using 15 *μ*L amplicons on the Nucleospin Gel and PCR Clean-up kit (Macherey-Nagel, Germany). The success of the clean-up process was verified by quantification with Qubit 2.0 ds DNA BR assay kit (Invitrogen, Eugene, USA) and on 1% agarose gel as described earlier. Sequencing reaction of each purified amplicon contained 60 ng amplicon, 2 *μ*L big dye 3.1, 0.5 *μ*M of forward or reverse primer, 1 *μ*L of 5x sequencing buffer, and sterile nuclease-free water to a final volume of 10 *μ*L. Sequencing was done bidirectionally using both forward and reverse primers in separate reactions. The sequencing conditions were denaturation at 96°C for 1 min, rapid ramp at 96°C for 10 s, rapid ramp at 56°C for 5 s for primer annealing, rapid ramp at 60°C for 4 mins and finally, and rapid ramp at 4°C for holding until purification. Sequencing PCR products were purified using sodium acetate (3 M, pH 4.6) and ethanol. The purified samples were kept frozen until they were sequenced. Partial 16S rRNA gene sequences were obtained using BigDye cycle sequencing on the ABI3500xl sequencer (Applied Biosystems, USA) of the Forestry and Agricultural Biotechnology Institute (FABI), University of Pretoria, South Africa. Sequences obtained were manually inspected using BioEdit (version 7.2.5, http://www.mbio.ncsu.edu/BioEdit/bioedit.html).

Preliminary identification of the isolates was done by aligning each sequence against sequences on the National Centre for Biotechnology Information (NCBI) GenBank using the basic alignment search tool, BLAST [25]. The mothur software pipeline [26] was used to cluster sequences into operational taxonomic units (OTUs) at sequence similarity of 97% or more. For taxonomic assignments, OTU representatives were aligned against sequences on the NCBI GenBank using BLAST. For phylogeny reconstruction, OTU representative sequences along with closely related sequences in the GenBank were selected for multiple sequence alignments using Mafft [27]. Multiple sequence alignments were edited and used to construct a neighbor-joining tree by using a maximum composite likelihood model and 1000-bootstrap replications in the MEGA6 [28].

## 3. Assessment of Soil Fertility Attributes

### 3.1. Phosphate Solubilization Assay

The ability of the isolated PAH-degrading bacteria to solubilize insoluble inorganic phosphate was investigated in the National Botanical Research Institute's Phosphate (NBRIP) growth medium [29]. The medium contains insoluble tricalcium phosphate (Ca_3_(PO_4_)_2_), as source of phosphate. Ten microliters of a 48-hour nutrient broth culture was dispensed into wells created on the medium and incubated at 30°C. Positive result for solubilization of phosphate was characterized by a clear halo around the inoculum well after 5–7 days of incubation. Phosphate solubilization index (PSI) describes the ability of the isolates to solubilize insoluble phosphate on culture plates; it is a ratio of the total diameter of the well and halozone to the diameter of the well [30].

(1) Phosphate  solubilization  index  PSI=well+diameter  of  halozonediameter  of  well.

### 3.2. Nitrogen-Fixation Assay

The Burks's nitrogen-free culture medium was used to screen isolates for atmospheric nitrogen-fixing ability. The appearance of appreciable growth within 7 days of aerobic incubation at 28°C was indicative of the isolates' nitrogen-fixing ability.

### 3.3. Indoleacetic Acid (IAA) Assay

The isolates were inoculated on a 1% tryptophan culture broth and incubated at 28°C for 48 hours with continuous agitation at 130 rpm. The culture broths were subsequently centrifuged at 10,000 rpm for 10 min at 4°C. Exactly 1 mL of the supernatant was mixed with 2 mL of Salkowsky's reagent (SR). The mixture was shaken and kept at prevailing ambient temperature in the dark for 30 min. The development of a pink coloration indicated the production of IAA. Subsequent quantification of IAA was done on a spectrophotometer at a wavelength of 540 nm [31]. A mixture of IAA and SR typically results in the formation of tris-(indole-3-acetato) iron (iii) complex which is displayed as the pink coloration. Therefore, IAA production was measured in the complex by a spectrophotometer and the obtained reading was used to calculate the IAA content by extrapolation from a standard curve of pure IAA.

### 3.4. Statistical Analyses

#### 3.4.1. Principal Component Analysis Comparison of Isolated Bacteria

A principal component analysis (PCA) was used to assess the ability of the isolates to perform soil fertility functions, which included solubilization of phosphate and production of indoleacetic acid, and the extent to which the isolates were different in their abilities to perform these. The principal component analysis was run in XLSTAT, Version 2015 (Addinsoft to Microsoft Excel 2013, New York, USA). The data was coded such that two principal ordination axes, which represented phosphate solubilization and indole acetic production, were extracted and biplots were plotted where arrow represented each function in the direction of its maximum change. Species were shown as points representing the capability of each one along that particular functional arrow. Long arrows indicate high capability for that function. Species occupying positions close to or beyond the tip of an arrow were deemed strongly positively correlated with and influenced by that function. Those at the opposite end were less strongly affected. The PCA model illustrates which species occur together and which ones are further apart in functional capability.

#### 3.4.2. Statistical Model Fitting

The bacteria were grouped with respect to the genus they belong to as* Pseudomonas*,* Rhodococcus*,* Arthrobacter*, and* Acinetobacter*. All other less prevalent bacteria isolates were placed in one category, others. A contribution to soil fertility was defined as a genus' ability to (i) produce indoleacetic acid, (ii) fix atmospheric nitrogen, or (iii) solubilize phosphorous. Generalized linear modelling techniques in SPSS version 21.0 were applied to assess whether each genus contributed significantly to enhancing soil fertility. For each genus, the first step was assessing whether the ability to produce indoleacetic acid, solubilize phosphorus, or fix atmospheric nitrogen was significantly different from just a neutral effect [32, 33]. Our models were compared against null models using the Likelihood Ratio Tests to judge if the specific contribution was significant. If significant, model fit (i.e., model adequacy) was assessed using the Wald Chi-square statistic [32, 33]. If model was adequate, assessment of which genus contributed most to the particular soil fertility function was evaluated. For this, ANOVA in conjunction with Tukey HSD Tests were applied.

## 4. Results and Discussion

### 4.1. Isolation, Identification, and Community Analyses of PAH-Degrading Bacteria

**Figure 1:**
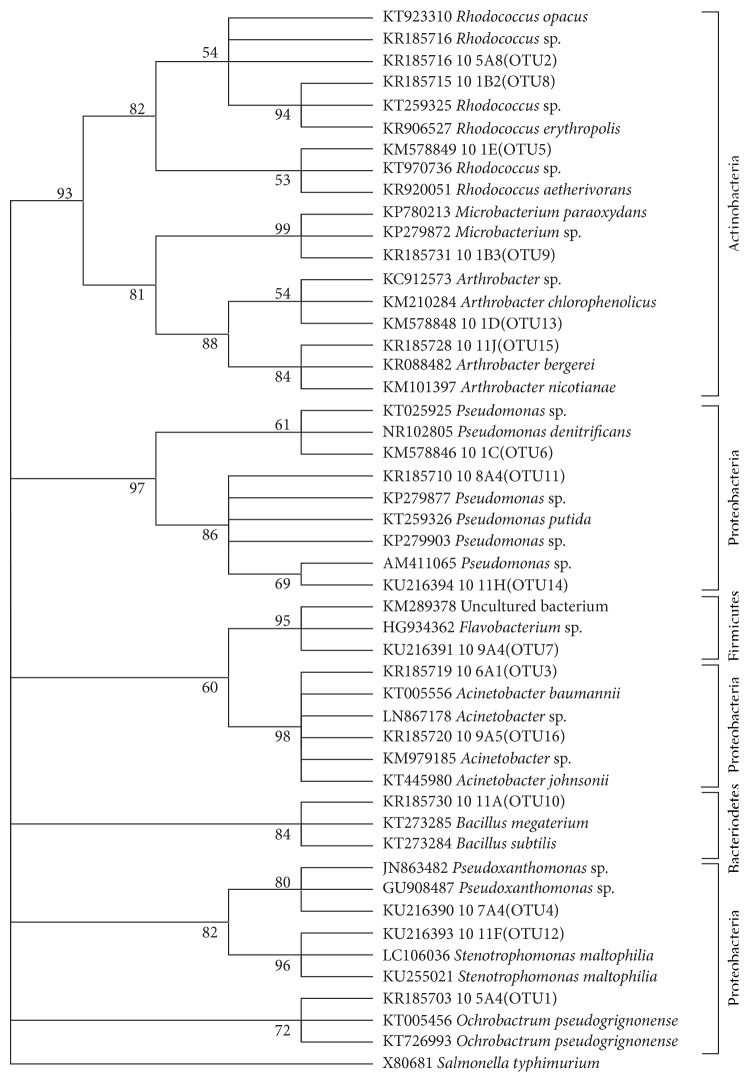
Evolutionary relationships of isolated soil bacteria. The evolutionary history was inferred using the neighbor-joining method. The percentage of replicate trees in which the associated taxa clustered together in the bootstrap test (1000 replicates) is shown next to the branches. The evolutionary distances were computed using the Jukes-Cantor method.* Salmonella typhimurium* was used as the outgroup. Phylogenetic analyses were conducted in MEGA6. One bacterial genus representing each OTU identified is depicted. The sequence analysis was carried out using mothur software in which 16 OTUs were generated at similarities of 97%. The relatives of the representative OTUs were obtained from NCBI. The nucleotides sequences of the representative OTUs showed a range of 98% to 100% identity with the homologous sequences reported in GenBank.

Forty-four PAH-degrading bacteria were isolated from enrichment cultures of simulated pollution soil treatments that were also biostimulated with vermicompost to add nutrients to the soil treatments [34]. Introduction of the pollutant could have slowed down the growth of indigenous soil bacteria but the biostimulation would have allowed the bacteria that could survive the pollution to thrive well. The isolated bacteria were those with PAH metabolism and degradation ability; they demonstrated varying levels of PAH degradation on culture plates, with a good number of them being Gram-negative. However, PAH-degradation potentials (details reported elsewhere) were independent of their Gram reaction. Sequences of the isolates clustered into 16 OTUs (Table 2). Taxonomic assignments revealed that the bacteria belonged to the phyla Proteobacteria, Firmicutes, Actinobacteria, and Bacteroidetes (Figure 1 and Table 2) and included the genera* Acinetobacter* (2 OTUs),* Arthrobacter* (2 OTUs),* Pseudomonas* (3 OTUs), and* Rhodococcus* (3 OTUs). The genera* Bacillus*,* Stenotrophomonas*,* Microbacterium*,* Pseudoxanthomonas*,* Ochrobactrum*, and* Flavobacterium* were also identified, with each of them occurring as either a single isolate or two isolates and clustering into a single OTU. Each of these genera of isolates have previously been associated with PAH degradation in different environmental matrices [14, 35, 36]. This study reported culturable PAH-degrading bacteria isolates. Both culturable and unculturable bacteria are known to be role players in PAH degradation [37]; in fact, reports are that only about 1% of total bacteria in soil are culturable [38]. This is no limitation to this study, as the focus of the study is to utilize the culturable isolates in field degradation experiments. Culturable* Pseudomonas* is also reported by Niepceron and coworkers [38] to be active degraders of naphthalene and phenanthrene in soil.

### 4.2. Soil Fertility Potentials Evaluation of Isolates

All of the isolates demonstrated potentials for at least one soil fertility potential, and many of them displayed multiple of such potentials.

#### 4.2.1. Phosphate Solubilization

**Figure 2:**
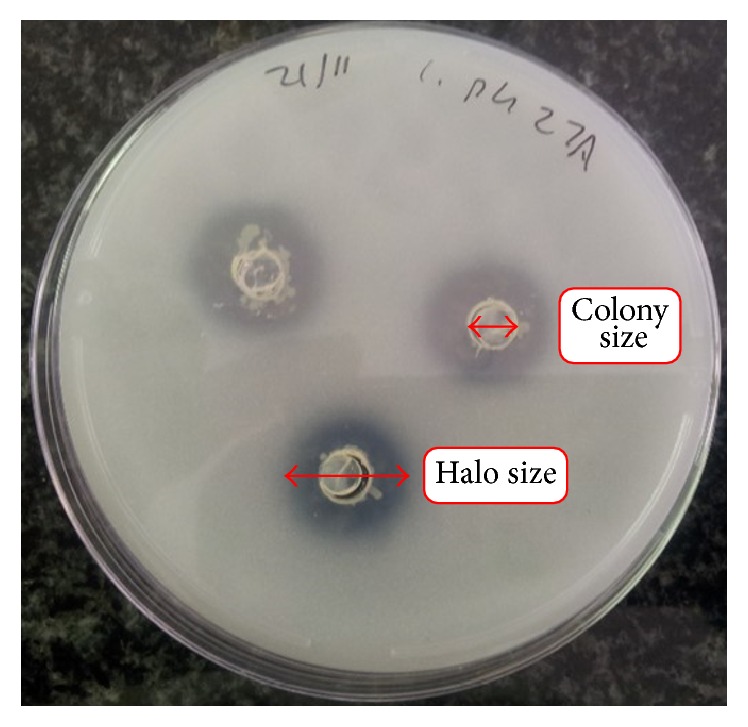
Plate showing phosphate solubilization colony size and halo size.

Thirty-one of the 44 isolates were able to solubilize insoluble calcium phosphate on culture plates. The isolates' solubilizing ability was measured as a mean value of the ratio of each halozone diameter to inoculum well diameter (Figure 2). This provided a good qualitative comparison of solubilization ability amongst the isolates. Phosphate solubilization index ranged from 2.17 (exhibited by an* Acinetobacter* sp.) to 7.28 (exhibited by a* Pseudomonas* sp.).* Pseudomonas* 1C and* Pseudomonas* 8A5 exhibited the highest PSI of 7.28 and 5.44, respectively; they both clustered into a single OTU suggesting that they could be very closely related. Phosphate solubilization indices from all the other isolates ranged from 2.17 to 3.44. Some other reports of phosphate solubilization by bacteria isolates are quantitative [39, 40] given in mg/L, when liquid culture media are utilized, which may or may not be associated with decrease in pH [40]. This present report of phosphate solubilization is given as indices; this may be quite relevant as an assessment of the relative abilities of the isolates with regard to how far from the source of inoculation the organisms can exert their ability. High phosphate solubilization ability was reported in endophytic* Pseudomonas* isolates where they stimulated the growth of* Pisum sativum* [9]. Phosphate solubilization and mineralization by soil bacteria have been deemed an important trait for bacteria to be used as soil inoculants to improve soil fertility and increase phosphate uptake by plants [41], phosphorus being the second major essential macronutrient for plant growth [4]. It is important in several plant metabolic processes such as photosynthesis and macromolecule biosynthesis as well as respiration [42]. Soil bacteria with phosphate solubilization attributes are therefore indispensable in enhancing soil fertility.

#### 4.2.2. Nitrogen-Fixation Ability

The isolates were rated as positive or negative depending on whether they displayed nitrogen fixation or not on culture plates. Thirty-eight isolates displayed growth on the culture plates, thus displaying potential for fixing atmospheric nitrogen, while 6 did not. The ability of the isolates to grow on a nitrogen-free culture medium is suggestive of the fact that the isolates are able to fix nitrogen in the atmosphere to facilitate their growth in a medium devoid of nitrogen, when indeed nitrogen is an essential nutrient for bacteria growth. Attribute of atmospheric nitrogen fixation by soil bacteria would be very important in improving soil fertility and imparting beneficial effects on the promotion of plant growth [43]. Despite the abundance of nitrogen in the atmosphere, plants are unable to use atmospheric nitrogen, unless first converted into utilizable forms such as ammonia and nitrates [44]. Fixation of atmospheric nitrogen, especially biological nitrogen fixation (BNF), would therefore be very essential for nitrogen to be useful to any organism [45]; it is estimated to contribute about half of yearly inputs of nitrogen to the terrestrial ecosystem [46].

#### 4.2.3. Indoleacetic Acid Production

The concentration of IAA produced by each isolate was extrapolated from a standard absorbance curve of different concentrations of pure IAA at wavelength of 540 nm. Forty-one of the isolates were able to produce IAA while 3 were not. The concentration of IAA produced ranged from 2.40 *μ*g/mL produced by* Acinetobacter* 11 M to 38.75 *μ*g/mL produced by* Bacillus* 11A.* Ochrobactrum* 5A4 produced the next highest quantity of IAA at a value of 33.90 *μ*g/mL while the next high quantities were produced by* Bacillus* 9A3 and* Pseudomonas* 4A1 at values of 29.40 *μ*g/mL and 22.70 *μ*g/mL, respectively.

### 4.3. Principal Component Analysis (PCA) of Isolates' Capabilities for Solubilization of Phosphate and Production of Indoleacetic Acid

**Figure 3:**
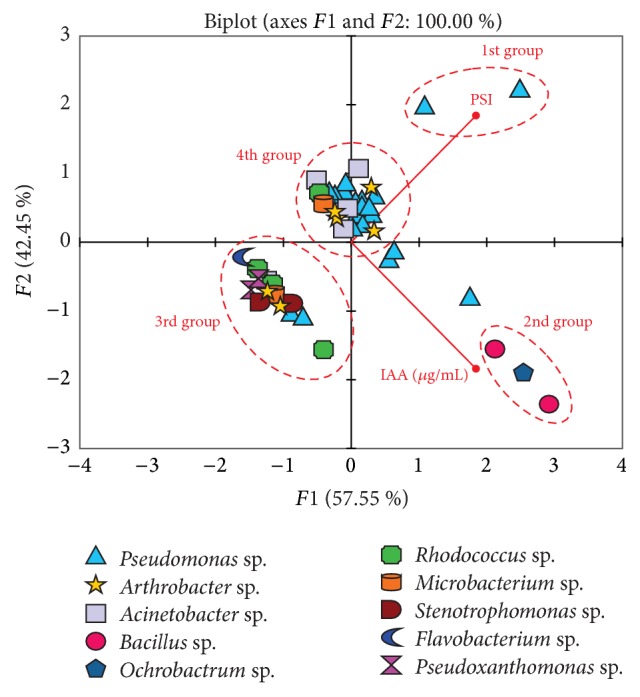
Principal component analysis (PCA) of the 44 isolates in relation to their abilities to solubilize phosphate (PSI) and produce indoleacetic acid (IAA) as a function of their potential ability to contribute to soil fertility.

Isolates' ability to enhance soil fertility via phosphate solubilization and IAA production was expressed on PCA (Figure 3). The first principal axis represents PSI and the second, almost of the same magnitude, represents IAA production. The PCA revealed four groups (designated 1st, 2nd, 3rd, and 4th group) of isolates. The 1st group consisted of 2* Pseudomonas* isolates that strongly correlated with PSI and 2nd group had 3 isolates strongly correlating with IAA. Inspection of results showed* Pseudomonas* 1C and* Pseudomonas* 8A5 exhibited the highest capability to solubilize insoluble tricalcium phosphate on culture plates while* Bacillus* 11A,* Ochrobactrum* 5A4, and Bacillus 9A3 were highest in indoleacetic acid production. A diverse group (3rd group) composed of the* Flavobacterium, Pseudoxanthomonas*, 3 isolates of the* Rhodococcus*, 2 isolates of the* Arthrobacter*, all 2 isolates of the* Stenotrophomonas*, 1 isolate of the* Acinetobacter*, 1 of the* Microbacterium*, and 2 isolates of the* Pseudomonas* grouped together, exhibiting weak or negative correlation with PSI. While the 4th group, also with diverse isolates including all 4* Acinetobacter* isolates, many of the* Pseudomonas*, 1 of the* Microbacterium, *4 of the* Arthrobacter*, and 1 of the* Rhodococcus*, were also weakly correlated with both IAA and PSI.

### 4.4. Evaluation of Phosphate Solubilization Ability of Isolates by Model Fitting

**Figure 4:**
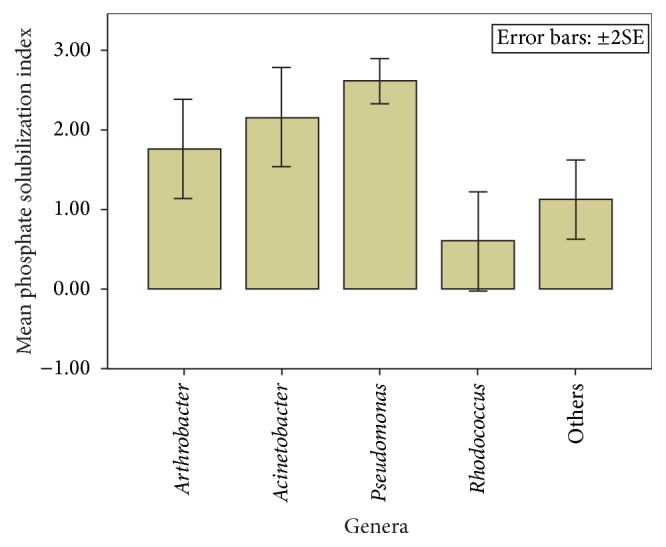
Phosphate solubilization indices of the groups of isolates.

Contributions of all the genera to solubilization of phosphate were different from 0 and the model was acceptable (Chi-square = 42.818, df = 4, and *P* = 0.000). The model exhibited adequate fit for data (Wald Chi-square = 26.186 and *P* = 0.00). There were differences in the ability to carry out phosphate solubilization amongst the bacterial genera. Pseudomonads exhibited the highest capability for this trait followed by* Acinetobacter* while* Rhodococus* exhibited the least capability (*F* = 12.66, *P* = 0.000, and df = 4) (Figure 4). The ability to solubilize phosphate was not significantly different between* Arthrobacter* and* Acinetobacter*. However, the ability to solubilize phosphate was higher for* Acinetobacter* and* Arthrobacter* compared to* Rhodococus* (*P* = 0.059) and (*P* = 0.008), respectively.

### 4.5. Evaluation of Nitrogen-Fixation Ability of Isolates by Model Fitting

The model with nitrogen fixation was not different from the null model (Chi-square is 1.832 df = 4, and *P* = 0.884) and the model fit was not plausible (Wald Chi-square is 1.146 and *P* = 0.887) implying the odds of fixing nitrogen for all genera were dependent on some other unknown factor, likely duration of incubation.

### 4.6. Evaluation of Indoleacetic Acid Production of Isolates by Model Fitting

**Figure 5:**
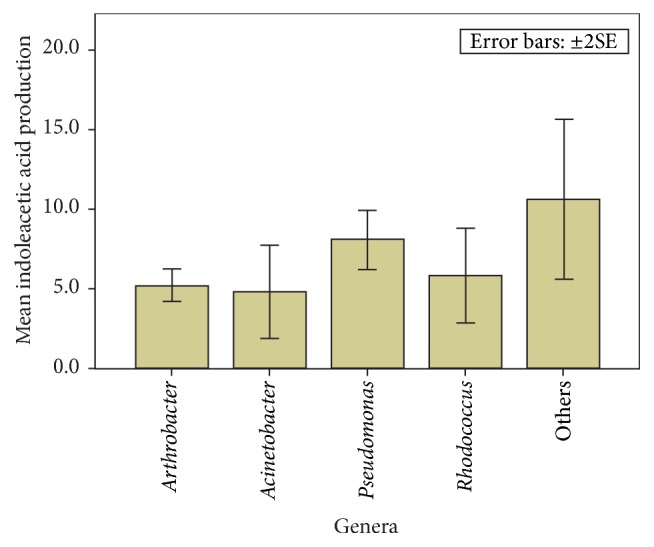
Indoleacetic acid production of the groups of isolates.

The Likelihood Ratio Chi-Square test was significant (Chi-square is 8.519, df = 4, and *P* = 0.008) implying that model was acceptable and that coefficients were different from the null model. The model had adequate fit (Wald Chi-square = 14.690, df = 4, and *P* = 0.005). Although Pseudomonads exhibited the highest ability to produce IAA, the differences amongst the genera for this trait were not significant (*F* = 1.991 and *P* = 0.100) (Figure 5).

This study identified different genera of bacteria with varying abilities to potentially contribute to soil fertility via phosphate solubilization, atmospheric nitrogen fixation, indoleacetic acid production, or a combination of any two or all three capabilities. Each of the genera exhibited the ability to potentially contribute significantly to soil fertility. Isolates in the genus* Pseudomonas* demonstrated the greatest ability to potentially contribute to soil fertility. Pseudomonads are known for being highly metabolically versatile in diverse ecosystems, including soil [47]. They have been reported as degrader strains in polluted soils [14, 48] as well as possessing several plant growth enhancing abilities [49, 50].

The isolates reported here were obtained from simulated PAH-polluted soil. Remediation processes that utilize plants as well as bacteria will find this useful as these isolates play an active role in remediation as well as enhance the growth of the plant involved in phytoremediation. Bacteria are important in the degradation of soil PAHs and are potentially useful in biorestoration of polluted sites [51]. An understanding of the potential capabilities of PAH-degrading bacteria in contributing to the fertility of soil is a pointer towards their usefulness in restoration of remediated land. Bacterial solubilization of phosphorus has been suggested to occur as a consequence of the bacteria releasing some short-chain organic acids [11] whose hydroxyl and carboxyl groups chelate cations bound to phosphate thereby making the phosphate soluble [52]. Short-chain organic acids were also found to inhibit the adsorption of some PAHs onto soil matrices while enhancing their desorption [53]. This shows that some products of metabolism may be responsible for the dual role exhibited by these isolates. It is also important to examine the metabolic genes possessed by the isolates for a better understanding of the isolates' abilities. The genome of one of the* Pseudomonas* isolates (*Pseudomonas* sp. 10-1B DDBJ/EMBL/GenBank; JYKS00000000) was sequenced, assembled, and annotated [54] and it provided an insight into the probable genes possessed by the isolates. Several phosphatases known to play an active role in phosphates solubilization and mineralization [43] were identified on the genome of the isolate. The gene for enzyme 1-aminocyclopropane-1-carboxylate (ACC) deaminase was also located on the genome. The presence of this enzyme has been reported in some plant growth promoting bacteria (PGPB) [55, 56]. The ACC deaminase enzyme enables bacteria to utilize ACC as the sole source of nitrogen by metabolizing it to ammonia and *α*-ketobutyrate [57]. Ethylene is known to prevent root elongation in plants and since ACC is the immediate precursor of ethylene metabolism of ACC therefore inhibits ethylene production and root elongation is promoted [56–59]. The possession of these genes by a typical bacterial isolate of this study suggests the suitability of these isolates to perform the dual role of biodegradation and soil fertility enhancement. It was observed from our results that all the degrading isolates could potentially contribute to soil fertility in at least one of the three attributes evaluated. Identification of species level could, perhaps, have explained the differences in their capabilities. These isolates can be further exploited to enhance soil fertility during phytoremediation. Lucy and coworkers [60] as well as Li and his coworkers [61] demonstrated that PGPB could be very useful as augmenting inoculum in phytoremediation.

## 5. Conclusion

The results of the present study suggest that the reported bacteria isolates have the potential of significant contribution to soil fertility while metabolizing pollutants. Isolates with such dual abilities will prove very useful in instances when sustainable and restorative remediation of polluted soil is desired and when there is need to increase agricultural sustainability in an environmentally friendly way. Such profound capabilities of bacteria exhibited in bioremediation and soil fertility are in high demand in the mining, in agroforestry sectors, and generally in sustainable agricultural production and environmental protection. The isolates in the genus* Pseudomonas* were most promising solubilizing phosphate; the* Bacillus* isolates and* Ochrobactrum* isolate did very well in their indoleacetic acid production. Further study will be undertaken to establish the isolates identity to species level, the metabolic products produced by them, and their genetic composition.

## Figures and Tables

**Table 1:** Some physical and chemical characteristics of the soil.

pH (H_2_O)	EC (mS/m)	Fe (mg/kg)	Cu (mg/kg)	Zn (mg/kg)	Total C (%)	Total N (%)	P Bray 1 (mg/kg)	CEC (cmol/kg)	Particle size (%)		
									Sand	Silt	Clay
7.60	0.52	49.72	2.09	3.63	0.232	0.31	28.93	13.917	76.0	8.0	16.0

**Table 2:** OTUs in relation to the phyla and the number of sequences they contain. The OTUs were computed using mothur software pipeline [26].

OTUs	Number of sequences	Accession numbers of sequences	Genus	Phylum
OTU 1	1	KR185703_10_5A4	*Ochrobactrum*	Proteobacteria
OTU 2	1	KR185716_10_5A8	*Rhodococcus*	Actinobacteria
OTU 3	2	KR185719_10_6A1, KR185718_10_3A2	*Acinetobacter*	Proteobacteria
OTU 4	1	KU216390_10_7A4	*Pseudoxanthomonas*	Proteobacteria
OTU 5	1	KM578849_10_1E	*Rhodococcus*	Actinobacteria
OTU 6	2	KM578846_10_1C, KR185712_10_8A5	*Pseudomonas*	Proteobacteria
OTU 7	1	KU216391_10_9A4	*Flavobacterium*	Bacteriodetes
OTU 8	2	KR185715_10_1B2, KR185714_10_1B1	*Rhodococcus*	Actinobacteria
OTU 9	2	KR185731_10_1B3, KR185732_10_7A3	*Microbacterium*	Actinobacteria
OTU 10	2	KR185730_10_11A, KR185729_10_9A3	*Bacillus*	Firmicutes
OTU 11	17	KR185710_10_8A4, KR185713_10_11C, KR185697_10_3A3, KR185709_10_8A3, KR185696_10_3A1, KR185701_10_4A4, KR185693_10_1A8, KR185695_10_2A3, KR185706_10_6A2, KR185699_10_4A2, KR185704_10_5A5, KM578847_10_1B, KR185700_10_4A3, KR185707_10_7A6, KR185702_10_5A3, KR185705_10_5A7, KR185698_10_4A1	*Pseudomonas*	Proteobacteria
OTU 12	2	KU216393_10_11F, KU216392_10_9A6	*Stenotrophomonas*	Proteobacteria
OTU 13	5	KM578848_10_1D, KR185727_10_11G, KR185726_10_11E, KR185725_10_11D, KR185723_10_7A2	*Arthrobacter*	Actinobacteria
OTU 14	1	KU216394_10_11H	*Pseudomonas*	Proteobacteria
OTU 15	1	KR185728_10_11J	*Arthrobacter*	Actinobacteria
OTU 16	3	KR185720_10_9A5, KR185722_10_11M, KR185721_10_11K	*Acinetobacter*	Proteobacteria

## Data Access

The sequences obtained have been deposited in the GenBank database with accession numbers KM578846-KM578849, KR185693-KR185707, KR185709, KR185710, KR185712-KR185716, KR185718-KR185723, and KR185725-KR185732.
